# Experimental sheep BSE prions generate the vCJD phenotype when serially passaged in transgenic mice expressing human prion protein

**DOI:** 10.1016/j.jns.2017.12.038

**Published:** 2018-03-15

**Authors:** Susan Joiner, Emmanuel A. Asante, Jacqueline M. Linehan, Lara Brock, Sebastian Brandner, Susan J. Bellworthy, Marion M. Simmons, James Hope, John Collinge, Jonathan D.F. Wadsworth

**Affiliations:** a MRC Prion Unit at UCL, UCL Institute of Prion Diseases, London, UK; b Animal and Plant Health Agency, Addlestone, Surrey, UK

**Keywords:** Bovine spongiform encephalopathy (BSE), Prions, Prion disease, Variant Creutzfeldt-Jakob disease (vCJD), Sheep-BSE, Transmissible spongiform encephalopathy (TSE)

## Abstract

The epizootic prion disease of cattle, bovine spongiform encephalopathy (BSE), causes variant Creutzfeldt-Jakob disease (vCJD) in humans following dietary exposure. While it is assumed that all cases of vCJD attributed to a dietary aetiology are related to cattle BSE, sheep and goats are susceptible to experimental oral challenge with cattle BSE prions and farmed animals in the UK were undoubtedly exposed to BSE-contaminated meat and bone meal during the late 1980s and early 1990s. Although no natural field cases of sheep BSE have been identified, it cannot be excluded that some BSE-infected sheep might have entered the European human food chain. Evaluation of the zoonotic potential of sheep BSE prions has been addressed by examining the transmission properties of experimental brain isolates in transgenic mice that express human prion protein, however to-date there have been relatively few studies. Here we report that serial passage of experimental sheep BSE prions in transgenic mice expressing human prion protein with methionine at residue 129 produces the vCJD phenotype that mirrors that seen when the same mice are challenged with vCJD prions from patient brain. These findings are congruent with those reported previously by another laboratory, and thereby strongly reinforce the view that sheep BSE prions could have acted as a causal agent of vCJD within Europe.

## Highlights

• Investigation of sheep BSE prions in transgenic mice expressing human prion protein.
• Observed molecular and neuropathological phenotypes are congruent with vCJD.
• Sheep BSE prions could have acted as a causal agent of vCJD within Europe.

## 1 Introduction

Bovine spongiform encephalopathy (BSE) is an epizootic transmissible spongiform encephalopathy (TSE) or prion disease of domestic cattle and causes variant Creutzfeldt-Jakob disease (vCJD) in humans following dietary exposure [1], [2], [3], [4]. Whilst the incidence of vCJD in the UK has been in decline for over a decade and the risk of new dietary exposure to BSE prions is remote, the majority of the UK population may have been exposed during the late 1980s and early 1990s. Because the interval between BSE prion exposure and development of clinical signs of vCJD may be decades [5], [6], [7] there is considerable uncertainty in knowing how many people may have been infected. Presently it is estimated that 1 in 2000 of the UK population may have subclinical prion infection [8] however the proportion of infected individuals that may go on to develop clinical disease, rather than remaining as asymptomatic carriers, is unknown [9].

Due to the inherent limitations of public health and epidemiological studies to address key uncertainties related to the nature and extent of BSE-related human prion disease in the UK, surrogate methods have been developed to evaluate the relative pathogenicity of animal prions for humans [10]. One major approach involves the experimental transmission of disease by inoculation of homogenised brain tissue from affected animals into transgenic mice overexpressing one or other of the two common polymorphic forms of the human prion protein (PrP) with either methionine (M) or valine (V) at residue 129 on a mouse PrP null background. Experimental transmission of BSE and vCJD prions to such mice has demonstrated the critical role of residue 129 polymorphism in determining susceptibility, incubation time and pathological phenotype (for review see Ref. [11]). These effects relate not only to the importance of homologous protein interactions in prion propagation [5], [12], [13], [14] but also to the preferential propagation of different prion strains by PrP with different primary structures via conformational selection [5], [11], [13], [14]. Findings from these models indicate that primary and secondary human infection with BSE prions may result in sporadic CJD-like or novel phenotypes in addition to vCJD, depending on the PrP genotype of the prion source and the recipient [4], [11], [15].

Because prion strains can adapt and mutate on passage in new species, and also within species as a result of PrP polymorphisms and other genetic factors, the evaluation of their risks to public health is complex [9], [11], [14], [16], [17], [18], [19]. Transgenic mouse models have therefore also been used to evaluate the zoonotic potential of prions generated by experimental transmission of cattle BSE prions to other species. Sheep and goats expressing different natural polymorphic variants of ovine or caprine PrP can be readily infected with BSE prions via the oral route (for recent review see Ref. [20]) and farmed animals were undoubtedly exposed to BSE-contaminated meat and bone meal. Discriminatory testing of all small ruminant TSE cases has been mandatory at EU level since 2005 (EC reg 999/2001 as amended, 36/2005) and while field cases of BSE-infected goats have been recognised [21], [22] retrospective studies within the UK have failed to identify any BSE-like cases in sheep [23], [24]. Nevertheless it cannot be ruled out that some TSE-affected sheep assigned at the time as cases of natural scrapie may in fact have been infected with BSE prions [25], particularly early in the epidemic before the feed ban to prohibit the inclusion of mammalian protein in animal feedstuffs was fully effective.

Two studies have previously concluded that experimental sheep BSE prions may propagate more efficiently than cattle BSE prions in transgenic mice expressing human PrP 129M [26], [27] and one of these studies convincingly established that sheep and goat BSE prions transmitted a molecular and neuropathological phenotype congruent with transmission of vCJD prions in the same mice [27]. These data strongly suggest that small ruminant BSE prions might act as a causal agent of vCJD [27]. Given the potential relevance of these findings to understanding the aetiology of vCJD in the UK (and as part of a larger transmission series evaluating the zoonotic potential of natural field cases of classical and atypical scrapie from sheep), we have also challenged our transgenic mice with isolates of experimental sheep BSE prions [28]. In contrast to the findings of others [26], [27], we observed a very low efficiency of transmission of experimental ovine BSE prions to mice expressing human PrP 129M and identified only a single subclinically infected mouse with detectable disease-related PrP (PrP^Sc^) in brain [28]. Despite this low attack rate (the potential reasons for which we have discussed previously [28]), we nevertheless considered it important to characterize the prion strain type that was generated from this transmission to inform upon whether vCJD prions or a novel prion strain type had propagated in our mice [28]. Here we now report the results from secondary passage of this isolate in further transgenic mice and in wild-type mice in comparison to the transmission properties of vCJD prions from patient brain to the same lines of mice.

## 2 Materials and methods

### 2.1 Biosafety

Work with prion-infected samples was conducted in microbiological containment level 3 or level 2 facilities with strict adherence to safety protocols.

### 2.2 Research governance

Storage and biochemical analysis of human tissue samples and transmission studies to mice were performed with informed consent from patients or relatives under approval from the Local Research Ethics Committee of UCL Institute of Neurology/National Hospital for Neurology and Neurosurgery and the code of practice specified in the Human Tissue Authority licence held by UCL Institute of Neurology. Work with animals was performed under licence granted by the UK Home Office (Project Licences 70/6454 and 70/7274) and conformed to University College London institutional and ARRIVE guidelines.

### 2.3 Transgenic and wild-type mice

Transgenic mice homozygous for a human PrP 129V transgene array and murine PrP null alleles (*Prnp*^*o/o*^), designated Tg(HuPrP129V^+/+^*Prnp*^*o/o*^)-152c mice (129VV Tg152c mice), or homozygous for a human PrP 129M transgene array and murine PrP null alleles (*Prnp*^*o/o*^), designated Tg(HuPrP129M^+/+^*Prnp*^*o/o*^)-35c mice (129MM Tg35c mice) have been described previously [28], [29], [30] and are fully susceptible to challenge with human prions. Both lines of mice have a congenic FVB/N, mouse PrP null, background and were derived from 129MM Tg35 and 129VV Tg152 parental lines which have been used extensively by us in previous human prion transmission studies [2], [4], [15], [31], [32]. 129MM Tg35c and 129VV Tg152c overexpress human PrP in brain at levels of 2- and 6-times that of pooled human brain, respectively. No spontaneous generation of prion infection has ever been observed in the parental (129MM Tg35 and 129VV Tg152) or the congenic (129MM Tg35c and 129VV Tg152c) lines of mice at advanced old age (either uninoculated groups of mice or recipients inoculated with vehicle only). Inbred FVB/NHsd mice (genotype *Prnp*^*a*^) were supplied by Harlan, UK.

### 2.4 Source of 129MM Tg35c-passaged ovine BSE prions

**Fig. 1:**
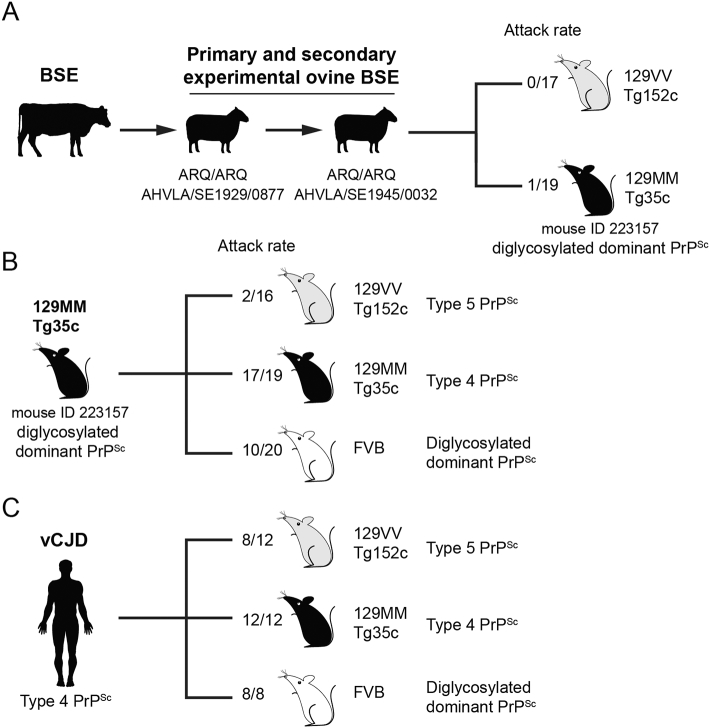
Summary of prion transmission rates to transgenic or wild-type mice. Mice were intracerebrally inoculated with 1% (w/v) brain homogenate. Attack rates report the total of clinically affected and subclinically infected mice as a proportion of the number of inoculated mice after prolonged post-inoculation periods. The type of PrP^Sc^ observed in the brain of affected mice is also reported. (A) Serial passage of cattle BSE prions in sheep (homozygous for ovine PrP with an ARQ genotype at codons 136, 154, 171) followed by transmission of sheep BSE prions to transgenic mice homozygous for human PrP with either methionine (M) or valine (V) at residue 129. These transmissions identified a single subclinically infected 129MM Tg35c mouse (ID 223157) that propagated a diglycosylated dominant PrP^Sc^ type in brain [28]. (B) Transmission of prions from mouse ID 223157 to further transgenic mice and to wild-type FVB mice. (C) Primary transmission of vCJD prions from patient brain (I336) to transgenic mice and to wild-type FVB mice. Full details of the transmissions shown in panels B and C are provided in the Tables.

Previously [28] we inoculated transgenic mice expressing human PrP (129MM Tg35c mice and 129VV Tg152c mice) with an experimental ovine BSE isolate (AHVLA SE1945/0032) that was obtained after secondary-passage of cattle BSE prions in sheep homozygous for ovine PrP with an ARQ genotype at codons 136, 154, 171 [33] (Fig. 1). Immunohistochemical and immunoblot analyses of brain following long post-inoculation survival periods identified a single subclinically affected 129MM Tg35c mouse that was culled 661 days post-inoculation when the experiment was terminated (mouse ID 223157) [28] (Fig. 1). PrP^Sc^ in the brain of this mouse showed a predominance of diglycosylated PrP and appeared similar to type 4 PrP^Sc^ (which is pathognomonic of vCJD [1], [34]) however the florid PrP plaques that characterize the propagation of the vCJD prion strain in humans [35] or transgenic mice [4] were not observed [28]. To define the prion strain type propagated in this transmission, brain inoculum from mouse ID 223157 was prepared by diluting 10% (w/v) brain homogenate to 1% (w/v) with Dulbecco's sterile phosphate buffered saline lacking Ca^2 +^ and Mg^2 +^ ions (D-PBS). Aliquots were stored frozen before inoculation into mice as described below.

### 2.5 Transmission of prions from mouse ID 223157

Mice (female, aged 6–8 weeks) were randomly assigned to experimental groups of 20 and anaesthetized with a mixture of halothane and O_2_, and intracerebrally inoculated into the right parietal lobe with 30 μl of 1% (w/v) brain homogenate from mouse ID 223157 prepared in D-PBS as described above. Thereafter all mice were examined daily for early indicators of clinical prion disease including piloerection, sustained erect ears, intermittent generalised tremor, unsustained hunched posture, rigid tail, mild loss of coordination, and clasping hind legs when lifted by the tail [30]. Definite diagnosis of clinical prion disease (triggering experimental end point) was reached if mice exhibited any two early indicator signs in addition to one confirmatory sign, or any two confirmatory signs. The confirmatory signs included ataxia, impairment of righting reflex, dragging of hind limbs, sustained hunched posture, or significant abnormal breathing [30]. Clinical diagnosis can be confounded by non-specific conditions which develop in mice as they age and there is considerable variation in the mean lifespans of different lines of transgenic mice and the onset of ageing artefacts. In this transmission series, we limited these confounding effects by electively culling mice after a defined post-inoculation period of 700 days. Such elective culling reduces the occurrence of ‘found dead’ mice that die of old age, in which brain tissue can undergo autolytic deterioration which precludes immunohistochemistry (IHC) analyses. At post-mortem, brains from inoculated mice were removed, divided sagittally with half frozen and half fixed in formol-saline.

### 2.6 Transmission of vCJD prions from patient brain

For more than twenty years we have conducted vCJD prion transmissions from the brain of multiple patients with neuropathologically confirmed vCJD. Initial studies used 129VV Tg152, 129MM Tg35 and wild-type FVB/N mice [2], [4], [15], [31], [32] while later studies have used 129MM Tg35c and 129VV Tg152c mice [28], [29] (and our unpublished data). In these transmissions 30 μl of 1% (w/v) brain homogenate (typically frontal cortex grey matter) prepared in sterile phosphate buffered saline was intracerebrally inoculated into mice after which mice were observed over their lifespan, or electively culled beyond 600 days post-inoculation. In the present study we have used these multiple historical vCJD prion transmissions as a control reference series and have used transmission data and mouse brain samples from one of these (vCJD patient brain isolate I336) to exemplify the distinguishing molecular and neuropathological phenotypes that are seen in the mice. Frozen brain homogenates and fixed brain samples from mice in these transmissions were re-analysed together with the new brain samples generated from the transmissions of brain inoculum from mouse ID 223157.

### 2.7 Immunoblotting

Frozen brain samples from mice or vCJD patients were prepared as 10% (w/v) homogenates in D-PBS using a tissue grinder [36]. Human or mouse brain homogenates were analysed by proteinase K digestion (50 or 100 μg/ml final protease concentration, 1 h, 37 °C) and immuno-blotting using high sensitivity enhanced chemiluminescence as described previously [36], [37]. Gels were calibrated using the Seeblue Pre-stained Protein Standard from Invitrogen. Detection of human PrP was performed using anti-PrP monoclonal antibody 3F4 [38] and the detection of mouse PrP was performed using ICSM 35 (D-Gen Ltd., London). Molecular strain typing of human PrP^Sc^ was performed using reference cases of human prion disease of known PrP^Sc^ type using the London classification [34]. Transgenic or wild-type mouse brain homogenates that were scored as negative for PrP^Sc^ after analysis of 10 μl 10% (w/v) brain homogenate were re-analysed by sodium phosphotungstic acid (NaPTA) precipitation of PrP^Sc^ from 250 μl of 10% (w/v) brain homogenate as described previously [37].

### 2.8 Neuropathology and immunohistochemistry

Brain fixed in 10% buffered formol-saline was immersed in 98% formic acid for 1 h and then embedded in paraffin wax. Serial sections (4 μm thick) were pre-treated by boiling for 10 min in a low ionic strength buffer (2.1 mM Tris, 1.3 mM EDTA, 1.1 mM sodium citrate, pH 7.8) before exposure to 98% formic acid for 5 min. Abnormal PrP accumulation was detected using anti-PrP monoclonal antibody ICSM 35 (D-Gen Ltd., London) on an automated IHC staining machine (Ventana Medical Systems Inc., Tucson, Arizona) using proprietary secondary detection reagents (Ventana Medical Systems Inc.) before development with 3′3 diaminobenzedine tetrachloride as the chromogen [36]. Conventional methods were used for Harris haematoxylin and eosin (H&E) staining. Positive controls for the staining technique were used throughout. All slides were digitally scanned on a LEICA SCN400 instrument, and images were captured from the LEICA slidepath software and composed with Adobe Photoshop. Abnormal PrP deposition in vCJD brain is distinguished by the presence of abundant florid PrP plaques consisting of a round amyloid core surrounded by a ring of spongiform vacuoles [35], [36]. The vacuolation pattern that characterises florid plaques is most clearly observed on H&E stained sections [35], [36].

## 3 Results and discussion

### 3.1 Summary of transmissions of vCJD prions to transgenic and wild-type mice

Over the last twenty years we have transmitted vCJD prions from multiple vCJD patient brain samples to transgenic mice expressing human PrP and wild-type FVB/N mice. All of the vCJD isolates we have examined behaved consistently in each line of mice with prion transmission properties that readily distinguish the vCJD prion strain from all other forms of human prion disease. In the present study we have used these multiple historical vCJD prion transmissions as a control reference series with which to compare our new transmissions and have used transmission data and mouse brain samples from one of these (vCJD patient brain isolate I336) (Fig. 1, Table 1) to exemplify the characteristic molecular and neuropathological phenotypes that are seen in the mice. In all of these studies (referenced below) we have never observed the spontaneous generation of prion infection in control groups of either transgenic or wild-type mice.

**Table 1:** Primary transmission of vCJD prions from patient brain to transgenic and wild-type mice.

Mouse line	Clinical attack rate^a^	Incubation period (days ± SEM)	Total attack rate^b^	Survival (days)^c^
129MM Tg35c	11/12	662 ± 12	12/12	548, 651–712 (11)
129VV Tg152c	1/12	719	8/12	460, 490, 517, 651–891 (9)^d^
FVB/N	6/8	342 ± 31	8/8	253, 260, 337, 355, 363, 403, 421, 426^e^

a All mice were inoculated with 30 μl of 1% (w/v) vCJD brain homogenate (code I336). Clinical attack rate is defined as the total number of clinically affected mice as a proportion of the number of inoculated mice. Incubation periods are reported for clinically affected mice in days; where n ≥ 3 the mean ± SEM is reported otherwise individual incubation times are given.

b Total attack rate is defined as the total number of clinically affected and subclinically infected mice as a proportion of the number of inoculated mice. Subclinical prion infection was assessed by immunohistochemical examination of brain for abnormal PrP deposition and immunoblot analysis of brain homogenate for PrP^Sc^.

c The interval between inoculation and culling (due to clinical prion disease, inter-current illness, senescence, or termination of the experiment) in days. Death dates of individual mice are shown together with the range for mice surviving beyond 600 days with the number of mice in this range shown in parentheses.

d Unaffected Tg129VV Tg152c mice were culled at 460, 490, 517 and 687 days post-inoculation.

e Subclinically infected FVB mice were culled at 363 and 403 days post-inoculation.

**Fig. 2:**
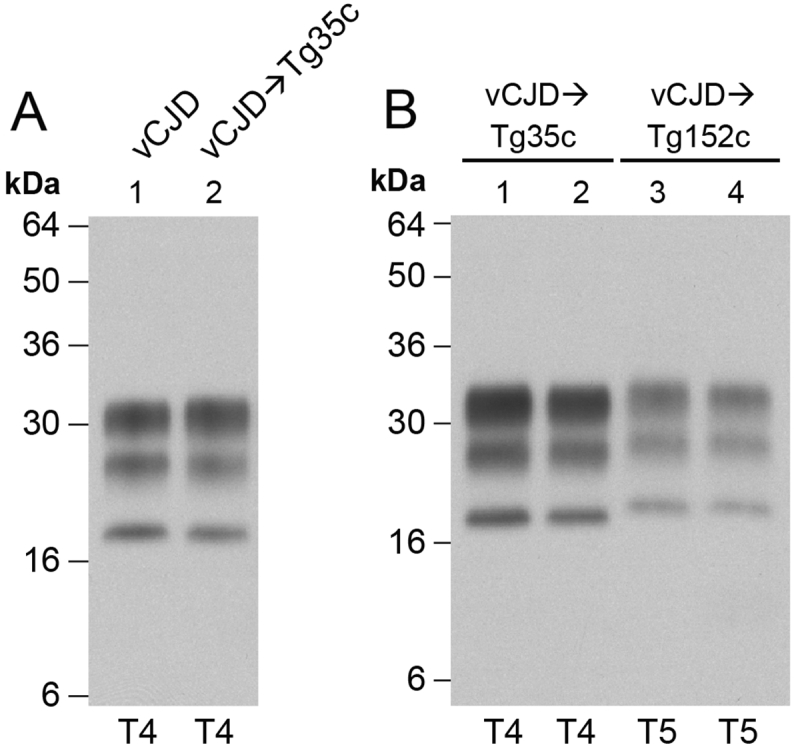
Molecular strain typing of vCJD prion transmissions from patient brain to transgenic mice. (A, B) Immunoblots of proteinase-K digested 10% (w/v) brain homogenates from a vCJD patient brain or vCJD prion-inoculated transgenic mice analysed by enhanced chemiluminescence with anti-PrP monoclonal antibody 3F4. The volumes of samples loaded were varied to give comparable levels of total PrP signal intensity in each lane. (A) vCJD patient brain and a clinically affected vCJD prion-inoculated 129MM Tg35c mouse that was culled 664 days post-inoculation demonstrating faithful propagation of type 4 PrP^Sc^ (T4). (B) Lanes 1 and 2, vCJD prion-inoculated 129MM Tg35c mouse brain from two clinically affected mice (culled 688 and 664 days post inoculation) showing propagation of type 4 PrP^Sc^ (T4) compared to brain from two subclinically infected vCJD prion-inoculated 129VV Tg152c mice (culled 860 and 716 days post-inoculation) propagating type 5 PrP^Sc^ (T5). Immunohistochemical analyses of brain from the same mice shown in panel B lanes 2 and 4 are presented in Fig. 3.

**Fig. 3:**
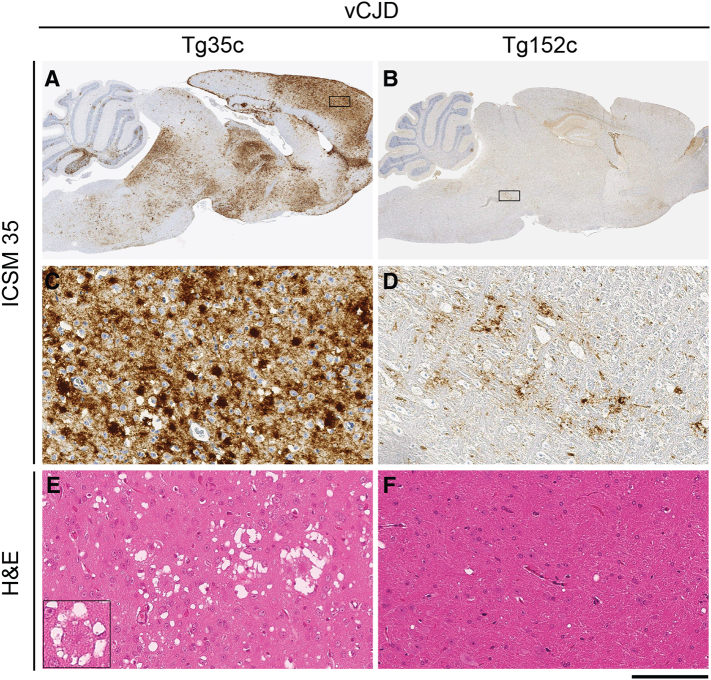
Neuropathological analyses of vCJD prion transmissions from patient brain to transgenic mice. Panels A, C, and E, a clinically affected vCJD prion-inoculated 129MM Tg35c mouse propagating type 4 PrP^Sc^ (see Fig. 2B, lane 2) culled 664 days post-inoculation. Panels B, D, and F, a subclinically infected vCJD prion-inoculated 129VV Tg152c mouse propagating type 5 PrP^Sc^ (see Fig. 2B, lane 4) culled 716 days post-inoculation. (A, B) Sagittal sections of whole brain. (C–F) Higher power magnification of the boxed regions shown in panels A and B; (C) cortex, (D) midbrain. (A–D) Abnormal PrP immunoreactivity stained with anti-PrP monoclonal antibody ICSM 35. (E, F) Haematoxylin- and eosin-stained sections (H&E) showing spongiform neurodegeneration including florid plaques in vCJD prion-inoculated 129MM Tg35c mouse brain (inset). Scale bars: A and B, 2 mm, C–F main panels 100 μm, inset panel E, 50 μm.

Challenge of our transgenic mice expressing human PrP 129M with vCJD prions from patient brain results in a high incidence of prion infection and faithful propagation of type 4 PrP^Sc^ (London classification; Fig. 1, Fig. 2, Table 1) [4], [15], [29], [31], [39] which is pathognomonic of the vCJD prion strain [1], [34]. Propagation of type 4 PrP^Sc^ in these mice is often accompanied by the key neuropathological hallmark of vCJD, the presence of abundant florid PrP plaques, which are frequently seen on a strong background of synaptic PrP deposition (Fig. 3) [4], [15], [29], [31], [39].

Notably, although vCJD prions produce high attack rates of infection in human PrP 129M transgenic mice (typically 100%) many affected mice do not develop clinical signs of prion disease and instead remain subclinically infected to advanced old age [4], [15], [29], [31], [39]. This lack of clinical end point has been observed with numerous vCJD brain isolates that we have examined, including those with very high levels of type 4 PrP^Sc^, and indeed, even with vCJD isolates that produce a high incidence of clinical prion disease the mean incubation periods are close to the lifespan of the mice. For example, although vCJD brain isolate I336 produced clinical prion disease in 11/12 inoculated 129MM Tg35c mice (which is the highest incidence we have so far observed) the mean incubation period was 662 ± 12 days (Table 1). Similar long mean incubation periods (around 500 days) for primary transmission and secondary passage of vCJD prions have also been seen by other researchers in Tg650 transgenic mice that express human PrP 129M in brain at 3-times higher levels than our 129MM Tg35c mice [27], [40]. This situation of prominent subclinical infection and highly prolonged clinical incubation times has effectively precluded our ability to reliably estimate vCJD prion titre in human PrP 129M mice using conventional serial dilution and incubation period methods [41]. As a consequence of this situation, we have found that demonstration of vCJD prion transmission to human PrP 129M mice is most reliably determined by demonstrating the propagation of type 4 PrP^Sc^ in the brain of inoculated recipients by immunoblotting rather than measuring the incidence and timing of clinical prion disease [4], [15], [39], [42]. Using this approach we have previously demonstrated the ability to detect prion transmission from a vCJD peripheral tissue containing PrP^Sc^ at a level 10^4.7^-fold lower than in the brain of the same vCJD patient and showed that propagating PrP^Sc^ in the brain of the recipient mice can be detected at an early stage of brain pathogenesis, well before abnormal PrP deposition becomes detectable by IHC [42]. While this study firmly established that the presence of minute quantities of PrP^Sc^ in vCJD tissues is indicative of the presence of infectious prions, it is important to note that PrP^Sc^ concentration may only broadly inform upon infectious prion titre as the majority of disease-related PrP present in vCJD brain is degraded by proteinase K [43], [44] and the proportional contribution of classical PrP^Sc^ to total infectivity remains unclear [45]. Difficulties in accurately correlating levels of detectable protease-resistant PrP with prion titre are well documented in other prion strain/host combinations [46] and indeed prion transmissions from tissues with undetectable protease-resistant PrP have been reported [47], [48].

In contrast to the efficiency with which vCJD prions infect human PrP 129M mice, primary challenge of transgenic mice expressing human PrP 129V is characterised by a substantial transmission barrier with only a proportion of inoculated mice becoming infected (Table 1, Fig. 1) [2], [15], [32], [49]. As with human PrP 129M mice, subclinical infection at advanced old age is a common feature of these transmissions. Affected vCJD-challenged human PrP 129V mice propagate a novel prion strain associated with type 5 PrP^Sc^ (Fig. 2) [2], [15], [32] which shares the same predominance of the diglycosylated PrP glycoform seen in type 4 PrP^Sc^ but is distinguished by proteinase K digestion products of greater molecular mass, indicative of a distinct PrP^Sc^ conformation (Fig. 2) [2], [15], [32]. Propagation of type 5 PrP^Sc^ following primary transmission of vCJD prions is generally associated with low levels of pathological PrP deposition in brain when visualised by IHC. Abnormal PrP deposition (when detected at primary transmission) is observed as focal or patchy diffuse labelling mainly in the midbrain and brainstem (Fig. 3) [2] and occasionally in some mice, as large non-florid PrP plaques in the corpus callosum [32]. There is a notable absence of florid PrP plaques even after secondary passage of type 5 PrP^Sc^ isolates in further human PrP 129V mice [15]. As with human PrP 129M mice, the most reliable way of assessing vCJD prion transmission rates in human PrP 129V mice is through detection of PrP^Sc^ in the brain of inoculated recipients following long clinically silent survival periods.

**Fig. 4:**
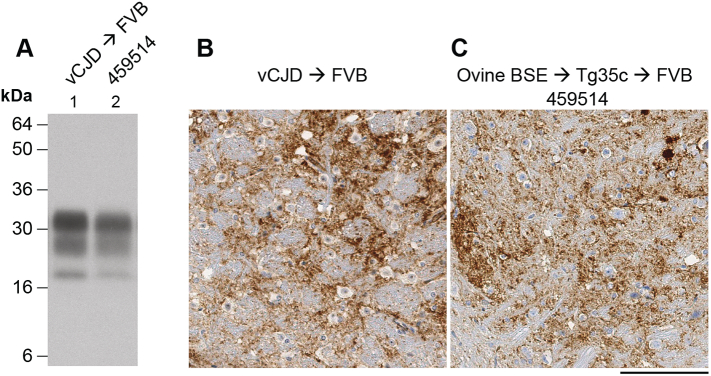
Immunoblot and immunohistochemical analyses of prion transmissions to wild-type FVB/N mice. (A) Immunoblot of proteinase-K digested 10% (w/v) brain homogenates analysed by enhanced chemiluminescence with anti-PrP monoclonal antibody ICSM 35. Lane 1, clinically affected FVB/N mouse inoculated with vCJD prions from patient brain culled 253 days post-inoculation. Lane 2, brain from a clinically affected FVB/N mouse (ID 459514) inoculated with 129MM Tg35c-passaged ovine BSE prions culled 603 days post-inoculation. (B, C) Deposition of abnormal PrP in the midbrain of clinically affected FVB/N mice inoculated with either vCJD prions from patient brain (B) or 129MM Tg35c-passaged ovine BSE prions (C) stained with anti-PrP monoclonal antibody ICSM 35. Scale bar; 100 μm.

In wild-type FVB/N mice, vCJD prions from patient brain have a high primary transmission rate, often resulting in clinical prion disease in affected mice, although incubation periods are prolonged (typically in the range of 300–400 days) (Table 1, Fig. 1) [2], [15], [32], [49]. In these transmissions a distinctive diglycosylated PrP dominant PrP^Sc^ type is propagated in brain (Fig. 4) which is identical to that seen after transmission of cattle BSE prions to the same mice [2], [15], [32]. Abnormal PrP deposition in affected mice consists mainly of patchy diffuse and granular deposits in the midbrain and brainstem with occasional small PrP plaques, however florid PrP plaques are not observed (Fig. 4) [2]. The efficiency with which vCJD prions transmit infection to wild-type FVB/N mice is remarkable and readily distinguishes the vCJD prion strain from all alternative prion strains that are propagated in other forms of human prion disease [2], [15], [32], [49].

### 3.2 Transmission of 129MM Tg35c-passaged ovine BSE prions to transgenic and wild-type mice

We generated brain inoculum from the single subclinically infected ovine BSE-challenged 129MM Tg35c mouse (mouse ID 223157) from our earlier study [28] (Fig. 1; see Materials and Methods) and inoculated this intracerebrally into further groups of 129MM Tg35c, 129VV Tg152c and FVB/N mice. The transmission properties of prions from this isolate in both transgenic mice and wild-type mice were entirely consistent with those of vCJD prions (Fig. 1, Table 2).

**Table 2:** Transmission of 129MM Tg35c-passaged ovine BSE prions in transgenic and wild-type mice.

Mouse line	Clinical attack rate^a^	Incubation period (days ± SEM)	Total attack rate^b^	Survival (days)^c^
129MM Tg35c	2/19	513, 690	17/19	493, 513, 527, 598, 616–701 (15)^d^
129VV Tg152c	2/16	590, 688	2/16	449, 458, 579, 588, 590, 598, 616–701 (10)
FVB/N	7/20	561 ± 38	10/20	410, 431, 515, 529, 532, 580, 585, 603–701 (13)^e^

a All mice were inoculated with 30 μl of 1% (w/v) brain homogenate from 129MM Tg35c mouse ID 223157 (see Fig. 1). Clinical attack rate is defined as the total number of clinically affected mice as a proportion of the number of inoculated mice. Incubation periods are reported for clinically affected mice in days; where n ≥ 3 the mean ± SEM is reported otherwise individual incubation times are given.

b Total attack rate is defined as the total number of clinically affected and subclinically infected mice as a proportion of the number of inoculated mice. Subclinical prion infection was assessed by immunohistochemical examination of brain for abnormal PrP deposition and immunoblot analysis of brain homogenate for PrP^Sc^.

c The interval between inoculation and culling (due to clinical prion disease, inter-current illness, senescence, or termination of the experiment) in days. Death dates of individual mice are shown together with the range for mice surviving beyond 600 days with the number of mice in this range shown in parentheses.

d Affected mice were culled at 493, 513, 527, 598 and between 616–701 days post-inoculation.

e Affected mice were culled at 410, 431, 532, 580 and between 603–701 days post-inoculation. Mice with clinical prion disease were culled at 410, 431, 580, 603, 610, 627 and 669 days post-inoculation.

**Fig. 5:**
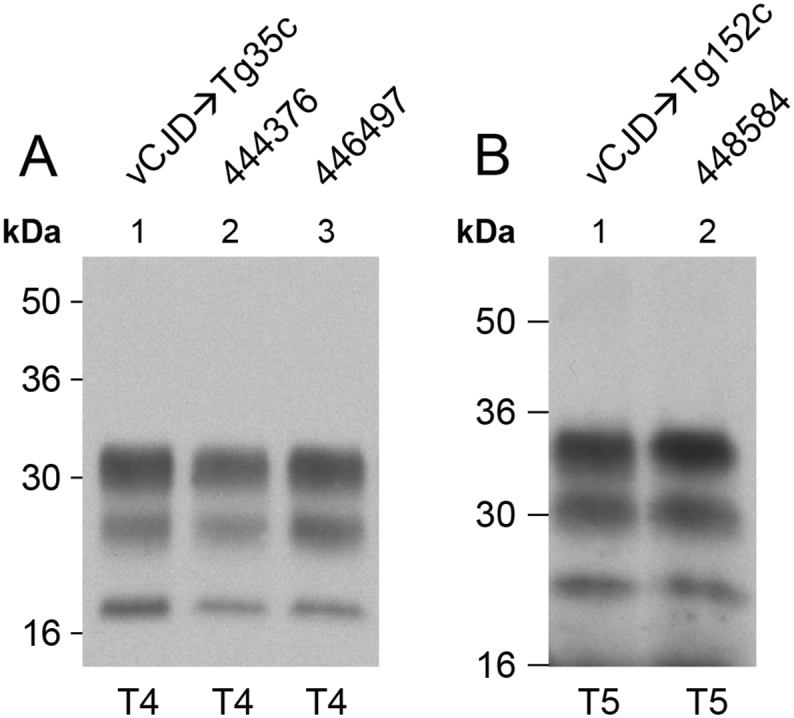
Molecular strain typing of 129MM Tg35c-passaged ovine BSE prion transmissions to transgenic mice. (A, B) Immunoblots of proteinase-K digested 10% (w/v) brain homogenates analysed by enhanced chemiluminescence with anti-PrP monoclonal antibody 3F4. The volumes of samples loaded were varied to give roughly equivalent levels of total PrP signal intensity in each lane. (A) Brain from a clinically affected 129MM Tg35c mouse inoculated with vCJD prions from patient brain (culled 664 days post-inoculation) is compared with brain from two subclinically affected 129MM Tg35c mice inoculated with 129MM Tg35c-passaged ovine BSE prions (ID numbers 444376 and 446497, both culled 701 days post-inoculation). All three brain samples show the propagation of type 4 PrP^Sc^ (T4). Immunohistochemical analyses of brain from the same mice shown in lanes 2 and 3 are presented in Fig. 6. (B) Brain from a subclinically infected 129VV Tg152c mouse inoculated with vCJD prions from patient brain (culled 687 days post-inoculation) is compared to brain from a clinically affected 129VV Tg152c mouse (ID number 448584) inoculated with 129MM Tg35c-passaged ovine BSE prions (culled 590 days post-inoculation). Both brain samples show propagation of type 5 PrP^Sc^ (T5).

**Fig. 6:**
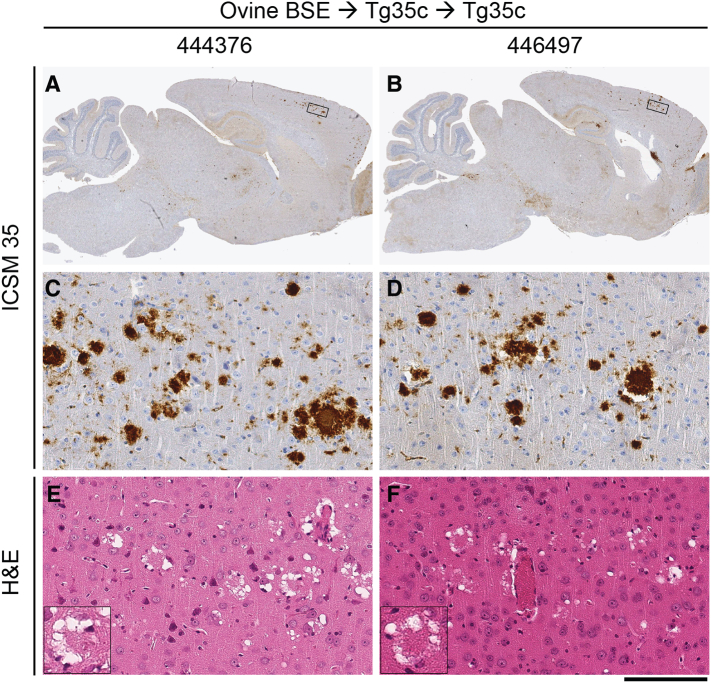
Neuropathological analysis of 129MM Tg35c-passaged ovine BSE prion transmissions to further 129MM Tg35c mice. Images show brain from two subclinically infected 129MM Tg35c mice inoculated with 129MM Tg35c-passaged ovine BSE prions; mouse ID numbers 444376 (panels A, C and E) and 446497 (panels B, D and F). Both mice propagated type 4 PrP^Sc^ (see Fig. 5A lanes 2 and 3) and were culled 701 days post-inoculation. (A, B) Sagittal sections of whole brain. (C–F) Higher power magnification of cortex from the boxed regions shown in panels A and B. (A–D) Abnormal PrP immunoreactivity stained with anti-PrP monoclonal antibody ICSM 35. (E, F) Haematoxylin- and eosin-stained sections (H&E) showing spongiform neurodegeneration including florid plaques (insets). Scale bars: A and B, 2 mm, C-F main panels 100 μm, inset in panels E and F, 50 μm.

In 129MM Tg35c mice we observed a high attack rate of infection (17 of 19 inoculated mice; Table 2) and the propagation of type 4 PrP^Sc^ (Fig. 5) in all affected mice. In mice in which abundant type 4 PrP^Sc^ was detected in brain homogenate (11 of 17 affected mice) numerous florid PrP plaques were observed in the cortex (Fig. 6). Although the incidence of clinical prion disease and the overall intensity of PrP deposition in brain were lower than seen after the transmission of vCJD prions from patient brain isolate I336 which we used as a comparator, this was well within the range we have observed from transmission of other vCJD prion isolates and probably simply reflects a lower prion titre in the inoculum. In this regard the PrP^Sc^ concentration in the brain of mouse ID 223157 was about 5% of that typically seen in vCJD patient brain homogenates [28]. As mentioned above, the frequent presence of subclinical infection in vCJD prion-inoculated human PrP 129M transgenic mice is not unusual, even when abundant abnormal PrP deposition and high levels of type 4 PrP^Sc^ are present in the brain of elderly mice [4], [15]. Interestingly, the two clinically affected mice seen after challenge with prions from mouse ID 223157 (Table 2) showed no detectable abnormal PrP deposition in brain by IHC (data not shown), although both were positive for type 4 PrP^Sc^ when brain was examined by immunoblotting.

Consistent with our findings in 129MM Tg35c mice, indicative of transmission of vCJD prions, inoculum from mouse ID 223157 transmitted poorly to 129VV Tg152c mice (Fig. 1, Table 2) with only 2 of 16 inoculated mice becoming infected (Table 2). Although the affected mice developed clinical disease after prolonged incubation periods and propagated type 5 PrP^Sc^ in the brain (Fig. 5), no abnormal PrP deposition was observed by IHC (data not shown), however, as discussed above, this is not unusual for primary transmission of vCJD prions to these mice.

In wild-type FVB/N mice, inoculum from mouse ID 223157 transmitted infection to 10 of 20 inoculated mice and produced clinical prion disease in 7 of the 10 affected mice. The mean incubation period in these mice was 561 ± 38 days (Table 2) (roughly 200 days longer than that typically seen with vCJD patient brain isolates (Table 1) [2], [15], [32]; indicative of a lower vCJD prion titre in the inoculum). All affected mice propagated the characteristic diglycosylated-dominant PrP^Sc^ type that is seen after transmission of vCJD prions from patient brain to the same mice (Fig. 4) [2], [15], [32]. Abnormal PrP deposition was observed in the brains of 2 of 5 affected mice available for IHC analyses, and consisted of patchy diffuse and granular deposits in the midbrain and brainstem which closely resembled the pattern seen after transmission of vCJD prions from patient brain to the same mice (Fig. 4).

## 4 Conclusions

Based upon the collective neuropathological and molecular phenotypes observed in the secondary transmission series reported here, we conclude that primary transmission of sheep BSE prions to 129MM Tg35c mice led to the propagation of the vCJD prion strain. These data concur with those of Torres and colleagues and strongly support their proposal that small ruminant BSE prions might act as a causal agent of vCJD [27]. This knowledge may have a future bearing upon understanding the zoonotic origin and epidemiology of vCJD and emphasizes the importance of continued discriminatory TSE surveillance of small ruminants within Europe [10], [25].

## Authors roles

JDFW directed the study and drafted the manuscript with SJ and MMS. EAA, JH and JC contributed to study design. SJB and MMS generated the original experimental sheep BSE isolates. EAA generated the transgenic mice. JDFW prepared inocula and coordinated the transmission study. SJ performed all the immunoblot analyses. JML, LB and SB carried out neuropathological analyses. All authors critically reviewed the data and contributed to and approved the final version of the manuscript. JDFW had full access to all the data in the study and had final responsibility for the decision to submit for publication.
